# Histone deacetylase 4 inhibits NF-κB activation by facilitating IκBα sumoylation

**DOI:** 10.1093/jmcb/mjaa043

**Published:** 2021-02-04

**Authors:** Qi Yang, Jielin Tang, Chonghui Xu, He Zhao, Yuan Zhou, Yanyi Wang, Min Yang, Xinwen Chen, Jizheng Chen

**Affiliations:** 1 Department of Gastroenterology, Guangzhou Women and Children’s Medical Center, Guangzhou 510623, China; 2 State Key Laboratory of Virology, Wuhan Institute of Virology, Center for Biosafety Mega-Science, Chinese Academy of Sciences, Wuhan 430071, China; 3 University of Chinese Academy of Sciences, Beijing 100049, China; 4 Guangzhou Institutes of Biomedicine and Health, Chinese Academy of Sciences, Guangzhou 510530, China

**Keywords:** histone deacetylase 4, sumoylation, IκBα, NF-κB, SUMO E3 ligase

## Abstract

Protein modification by small ubiquitin-like modifier (SUMO) is an important regulatory mechanism for multiple cellular processes. Although the canonical pathway involving the ubiquitylation or phosphorylation of IκBα has been well characterized, little is known about the sumoylation of IκBα in the control of NF-κB activity. Here, we find that histone deacetylase 4 (HDAC4) negatively regulates tumor necrosis factor-alpha- or lipopolysaccharide-triggered NF-κB activation. HDAC4 belongs to the SUMO E3 ligase family and can directly sumoylate IκBα. The cytoplasm location of HDAC4 is essential for IκBα sumoylation. The Cys292 of HDAC4 is a key site for its SUMO E3 ligase activity. The sumoylation of IκBα prevents its polyubiquitination and degradation because these two modifications occur both at the Lys21. Our findings reveal a previously undiscovered role for HDAC4 in the inflammatory response as a SUMO E3 ligase for IκBα sumoylation. Our work provides insight into mechanisms ensuring optimal mediation of the NF-κB pathway.

## Introduction

In humans and mice, the 18 histone deacetylase (HDAC) enzymes are grouped into four classes by both sequence similarity and enzymatic activity, including the classical HDACs (classes I, II, and IV) and nonclassical class III sirtuins. The transcriptional repression mediated through HDACs is a critical component of eukaryotic gene regulation. Recently, classical HDACs have been attributed key roles in innate and adaptive immune pathways, including epigenetic regulation of innate response (Choi et al., 2016; Li et al., 2016; Meng et al., 2016) and inflammation (Aung et al., 2006; Foster et al., 2007), as well as lymphocyte development and function (de Zoeten et al., 2010; Yamaguchi et al., 2010; Navarro et al., 2011). HDAC4 belongs to the HDAC family of class IIa, which interacts with members of the related MEF2-interacting transcription repressor protein (Miska et al., 1999) and with the 14-3-3 chaperone proteins in the control of myeloblast differentiation (Grozinger and Schreiber, 2000; McKinsey et al., 2000). Furthermore, HDAC4 can regulate MEF2 and LXRα sumoylation (Zhao et al., 2005; Lee et al., 2009), and key roles for HDAC4 in insulin sensitivity and energy balance have been identified recently (Luan et al., 2014).

Inflammation plays an important role in stress injury or infection with microbes. One hallmark following stimulation with the proinflammatory cytokines, such as tumor necrosis factor-alpha (TNFα) and interleukin-1β (IL-1β), is the activation of nuclear factor-kappa enhancer-binding protein (NF-κB) and mitogen-activated protein kinases, and the subsequent induction of cytokines and chemokines. In addition, inflammatory responses are tightly controlled to prevent excessive responses and chronic inflammatory states by positive and negative regulatory mechanisms and close interactions with other signaling pathways. Many diseases, including infectious diseases, autoimmune diseases, and cancers, show an intrinsic correlation with inflammatory disequilibrium caused by the misregulation of many cytokines and chemokines (Grivennikov et al., 2010; Harhaj and Dixit, 2011; Oeckinghaus et al., 2011).

The transcription factor NF-κB regulates diverse physiological and pathological processes, such as cell survival and proliferation, tumorigenesis, and immune and inflammatory responses. The mammalian Rel/NF-κB family of dimeric transcription factors includes five members, namely RelA (p65), RelB, c-Rel, p100/p52, and p105/p50. In resting cells, heterodimeric NF-κB proteins are sequestered in the cytoplasm as inactive subunits by inhibitory IκB proteins. After exposure of cells to activators, such as TNFα, IL-1β, or lipopolysaccharide (LPS), IκB is rapidly phosphorylated, ubiquitinated, and ultimately degraded by the 26S proteasome (Traenckner et al., 1995). The degradation of IκBα frees NF-κB to translocate into the nucleus, where binding of NF-κB to its DNA recognition sites activates the transcription of responsive genes involved in inflammation and survival (Baeuerle and Baichwal, 1997; Karin and Ben-Neriah, 2000; Chen et al., 2002). The active NF-κB is tightly controlled at multiple levels by many regulatory elements, all of which ensure transient NF-κB signaling in response to a stimulus. For instance, the deubiquitinase CYLD- and A20-mediated downregulation of NF-κB is of critical importance (Sun, 2008; Harhaj and Dixit, 2011).

IκBα is a member of the inhibitory IκB family, which also contains IκBβ, IκBγ, IκBε, p100, p105, and Bcl-3 (Karin and Ben-Neriah, 2000; Vallabhapurapu and Karin, 2009). Among all inhibitory IκB proteins, IκBα, IκBβ, and IκBγ are the most central regulators of mammalian NF-κB. All IκBs contain either six or seven ankyrin repeats, and helical domains mediate binding to the Rel homology region and masking of NF-κB. Only IκBα, IκBβ, and IκBε contain N-terminal regulatory regions, which are required for stimulus-induced degradation. In response to stimulation with proinflammatory cytokines, the IκBα gene is specifically induced by the p65 subunit of NF-κB using an inducible autoregulatory pathway (Sun et al., 1993). Excepting for the sequestration of NF-κB in the cytoplasm, newly synthesized IκBα plays an important role in the negative regulation of NF-κB-dependent transcription (Sosic et al., 2003). SUMO1 modification of IκBα also plays a crucial regulatory role, because it makes the protein resistant to signal-induced degradation by blocking ubiquitination and inhibits NF-κB activation (Desterro et al., 1998). However, there is considerable complexity in how IκBα is modified by SUMO1 and coordinates a nuanced NF-κB response.

In this report, we describe HDAC4 as a SUMO E3 ligase for negatively regulating IκBα in response to stimulation with TNFα or LPS. We found that Cys292 (C292) of HDAC4 is critical for SUMO E3 ligase activity, and IκBα is mainly modified with SUMO1 at Lys21 (K21). Our findings provide mechanistic insights into the regulation of IκBα stability and identify a previously unknown manner whereby HDAC4 controls IκBα sumoylation to prevent excessive responses and uncontrolled inflammation.

## Results

### The involvement of HDAC4 in proinflammatory cytokine production

**Figure 1:**
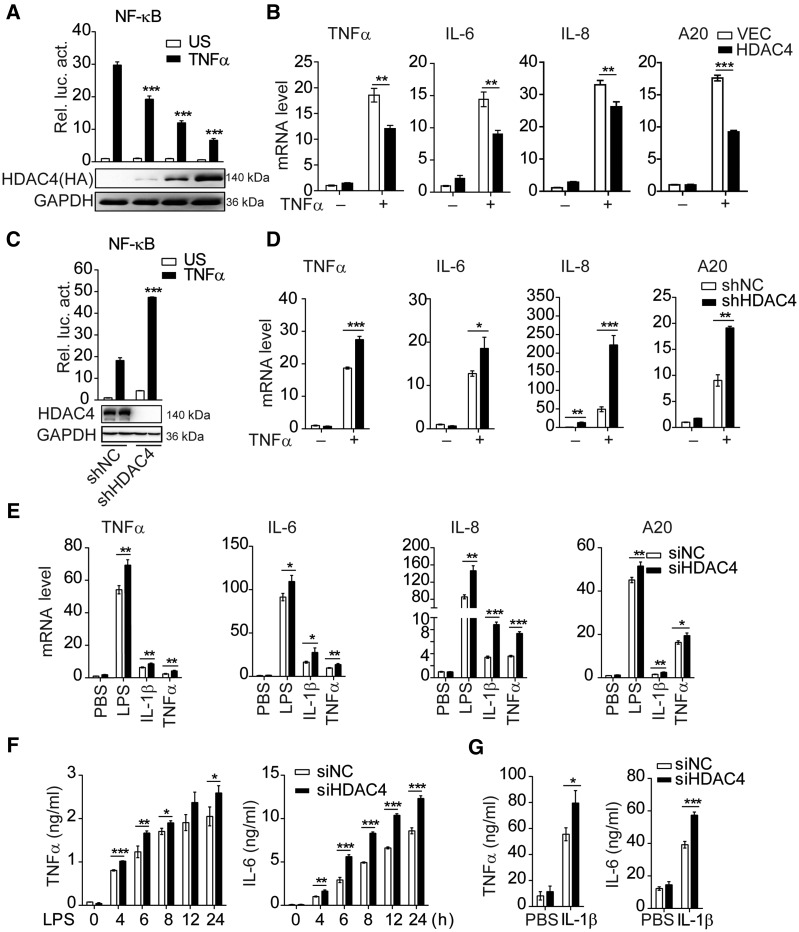
Identification of HDAC4 as a negative regulator of NF-κB signaling. (**A**) HDAC4 dose-dependently suppresses the TNFα-triggered activation of the NF-κB promoter in HEK293T cells. Cells (1 × 10^5^) were transfected with the NF-κB firefly luciferase reporter plasmid (0.01 µg) and URL-TK (Renilla luciferase as an internal control for transfection efficiency, 5 ng), as well as the indicated amount of a control (VEC, the empty vector) or HA-HDAC4 plasmid (0.1, 0.25, 0.5 µg). Twenty-four hours after transfection, cells were treated with TNFα (20 ng/ml) or untreated (US) for 10 h before luciferase assays were performed. The histogram shows the ratio of firefly luciferase values (NF-κB reporter) to Renilla luciferase values and normalized to the mock untreated cells. (**B**) Effect of HDAC4 (0.25 µg) on TNFα-induced transcription of TNFα, IL-6, IL-8, and A20 genes. (**C**) Effect of HDAC4 on TNFα-triggered NF-κB activation in HDAC4-stable knockdown HEK293T cells. The cells were transduced with either a control (shNC) or shHDAC4 plasmid to establish stable cell lines (shHDAC4). The cells (1 × 10^5^) from stable cell lines were transfected with the NF-κB reporter plasmid (0.01 μg) and pRL-TK (5 ng). Dual-luciferase assays were performed as in **A**. (**D**) Effect of HDAC4 knockdown on TNFα-induced transcription of downstream genes in HDAC4-stable knockdown HEK293T cells. The shHDAC4 and shNC HEK293T cells were treated with TNFα (20 ng/ml) for 10 h before real-time PCR analysis. Real-time PCR assays were performed as in **B**. (**E**) Effect of HDAC4 knockdown on LPS-, IL-1β-, or TNFα-induced transcription of downstream genes in BMDCs. The BMDCs were treated with control or HDAC4-specific siRNA for 48 h and stimulated with LPS (100 ng/ml), IL-1β (20 ng/ml), or TNFα (20 ng/ml) for 12 h before real-time PCR. (**F** and **G**) ELISA of TNFα and IL-6 in supernatants of HDAC4-knockdown BMDCs or the control with LPS (100 ng/ml) or IL-1β (20 ng/ml) for 12 h. Data are representative of three independent experiments. Graphs show mean ± SD; *n* = 3. *P < 0.05, **P < 0.01, ***P < 0.001.

In our previous work, we indicated that HDAC4 could mediate the activation of type I interferon signaling via a tetramer with TBK1/IKKε–HDAC4–IRF3 in the cytoplasm, and the interferon (IFN-β) could stimulate the HDAC4 expression (Yang et al., 2019). It has been reported that more TNFα was produced in HDAC4 macrophage-specific knockout mice compared with wild-type (WT) mice (Luan et al., 2014). This suggested that the endogenous HDAC4 is also involved in the regulation of NF-κB signaling. To confirm this hypothesis, we found that overexpression of HDAC4 dose-dependently inhibited TNFα-triggered activation of NF-κB in HEK293T cells and decreased NF-κB-related gene transcription, such as the transcription of TNFα, IL-6, IL-8, and A20 (Figure 1A and B). Knockdown of endogenous HDAC4 via shRNA or siRNAs potentiated the TNFα-triggered activation of NF-κB in reporter assays (Figure 1C; Supplementary Figure S1A). Moreover, the NF-κB-related genes were increased at different levels in HDAC4-knockdown HEK293T cells after TNFα stimulation (Figure 1D). However, knockdown of HDAC4 did not affect IFNγ-triggered IRF1 promoter activity and phosphorylation of STAT1 (Supplementary Figure S1B–D). We obtained the same results in mouse macrophage RAW264.7 (Supplementary Figure S2A–C). Furthermore, HDAC4 silencing with siRNA in bone marrow-derived dendritic cells (BMDCs) markedly upregulated the mRNA levels of TNFα, IL-6, IL-8, and A20 induced by LPS, IL-1β, or TNFα (Figure 1E). Consistently, TNFα and IL-6 were also significantly increased after different stimuli (Figure 1F and G). Collectively, these data suggest that HDAC4 negatively regulates NF-κB signaling.

### HDAC4 positively regulates the stability of IκBα

**Figure 2:**
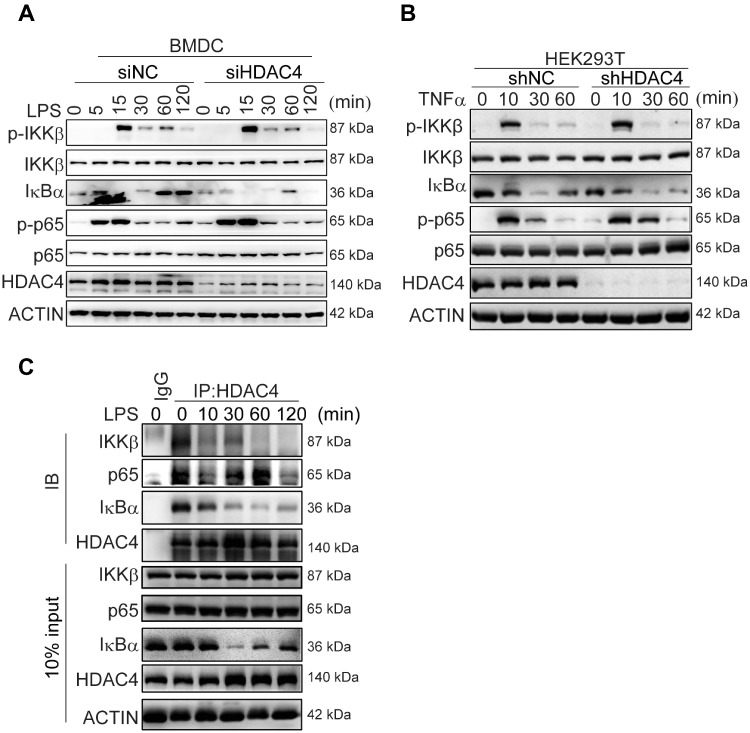
HDAC4 interacts with IκBα and p65. (**A** and **B**) Immunoblotting analysis of phosphorylated (p-) or total IKKβ, p65, and IκBα, HDAC4, or ACTIN (loading control throughout) during LPS or TNFα challenge in BMDCs or HEK293T cells. HDAC4 knockdown was performed by using siRNA or shRNA. (**C**) Endogenous interactions between HDAC4 and IKKβ, p65, or IκBα. BMDCs were left untreated or treated with LPS (100 ng/ml) and HEK293T cells were treated with TNFα (20 ng/ml) for the indicated times before coimmunoprecipitation (coIP) and immunoblotting (IB) analyses.

To understand the mechanism by which HDAC4 negatively regulates inflammatory signaling, we analyzed the major responses in the signaling pathways of BMDCs or HEK293T cells challenged with LPS or TNFα. In BMDCs, compared with the control group, the level of phosphorylated p65 induced by LPS increased more significantly upon HDAC4 silencing, especially in the early stage (5–15 min). Correspondingly, lower levels of IκBα were manifested in the early stage (5–15 min) and late stage (60–120 min) in the HDAC4-knockdown cells (Figure 2A). We obtained the same conclusion in HEK293T cells challenged with TNFα (Figure 2B). Then, we analyzed the interactions of IκBα, p65, or IKKβ with HDAC4 in BMDCs after stimulation with LPS. Endogenous HDAC4 was associated with IκBα in the resting BMDCs; the interaction between HDAC4 and IκBα rapidly decreased and finally disappeared after 10–30 min as IκBα Ser32/36 (S32/36) was phosphorylated and degraded after stimulation with LPS. New synthesis of IκBα appeared in 60–120 min, subsequently, the interaction between HDAC4 and IκBα increased. In addition, HDAC4 was also associated with IKKβ or p65 (Figure 2C). However, there was no effect on the phosphorylation of IKKβ in the HDAC4-knockdown cells stimulated with LPS or TNFα (Figure 2A and B). Thus, HDAC4 might target p65 or IκBα to regulate the NF-κB activation.

The acetylation of p65 at K310 is required for DNA binding activity of NF-κB (Chen et al., 2002). To explore whether the increased transactivation activity of NF-κB stems from its hyperacetylation in HDAC4-knockdown cells, we next tested the transcriptional activity and acetylation at K310 of p65 using the lysine-to-arginine substitution mutants of p65. Transactivation by the p65 K310R mutant was comparable with that of WT p65 in HDAC4-knockdown 293T cells (Supplementary Figure S3A). In addition, knockdown of HDAC4 did not interfere with the acetylation at K310 of p65 in HEK293T cells (Supplementary Figure S3B). A reporter assay indicated that Tasquinimod, a specific HDAC4 deacetylase activity inhibitor, could not rescue the inhibition of HDAC4 on the NF-κB transcriptional activity (Supplementary Figure S3C and D). Thus, the deacetylase activity of HDAC4 had little effect on its negative regulation of the NF-κB transcriptional activity.

**Figure 3:**
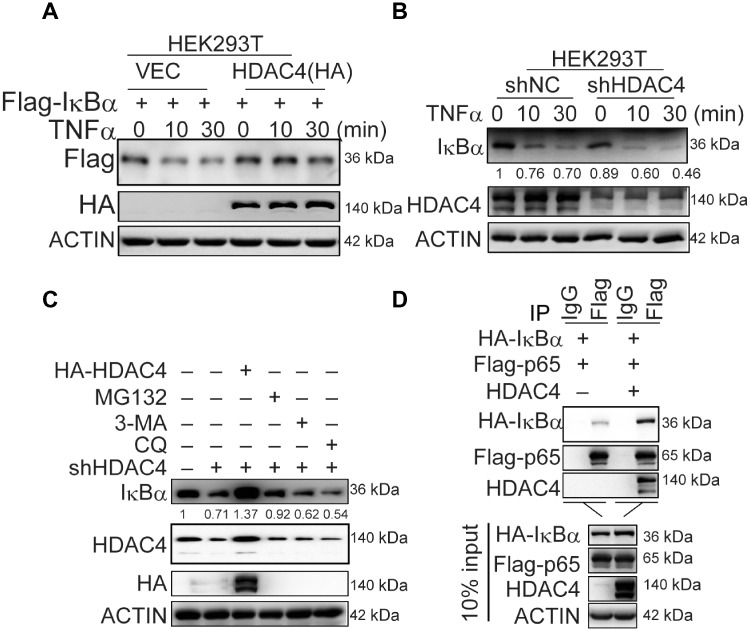
HDAC4 positively regulates the level of IκBα protein. (**A**) Effect of HDAC4 on the level of IκBα in HEK293T cells. Cells (4 × 10^5^) were transfected with the indicated plasmids for 36 h and then were treated with TNFα (20 ng/ml) for the indicated time or left untreated. The cells were lysed, and the lysates were analyzed by immunoblotting with the indicated antibodies. (**B**) Effect of HDAC4 knockdown on the level of IκBα proteins. The control or HDAC4-knockdown cells (1 × 10^5^) were treated with TNFα (20 ng/ml) for the indicated time or left untreated. (**C**) Effect of HDAC4 on the stability of IκBα. The HDAC4-knockdown cells (4 × 10^5^) were transfected with the indicated plasmids or treated with the indicated inhibitors MG132 (1 µg/ml), 3-MA (60 µM), and CQ (50 µM) for 4 h before immunoblotting analysis was performed. (**D**) HDAC4 enhances the interaction between IκBα and NF-κB. HEK293T cells (2 × 10^6^) were transfected with the indicated plasmids (2 µg each). Cell lysates were incubated with a Flag antibody. Bound proteins were analyzed by immunoblotting with anti-HA, anti-Flag, and anti-HDAC4. Data are representative of three independent experiments with similar results.

The knockdown of HDAC4 decreased the level of IκBα after LPS stimulation (Figure 2A). Thus, HDAC4 might regulate the stability of IκBα. We routinely found that IκBα was more resistant to TNFα-induced degradation, and HDAC4 markedly upregulated the level of IκBα in the overexpression system (Figure 3A). IκBα was more sensitive to TNFα-induced degradation in HDAC4-knockdown cells than in the control cells (Figure 3B). Furthermore, MG132 treatment or anaplerotic HDAC4 rescued the expression of IκBα in HDAC4-knockdown cells (Figure 3C). Moreover, the interaction between NF-κB and IκBα was enhanced by HDAC4 (Figure 3D). Taken together, these results indicated that HDAC4 could maintain the protein stability of IκBα.

### HDAC4 catalyzes the sumoylation of IκBα

**Figure 4:**
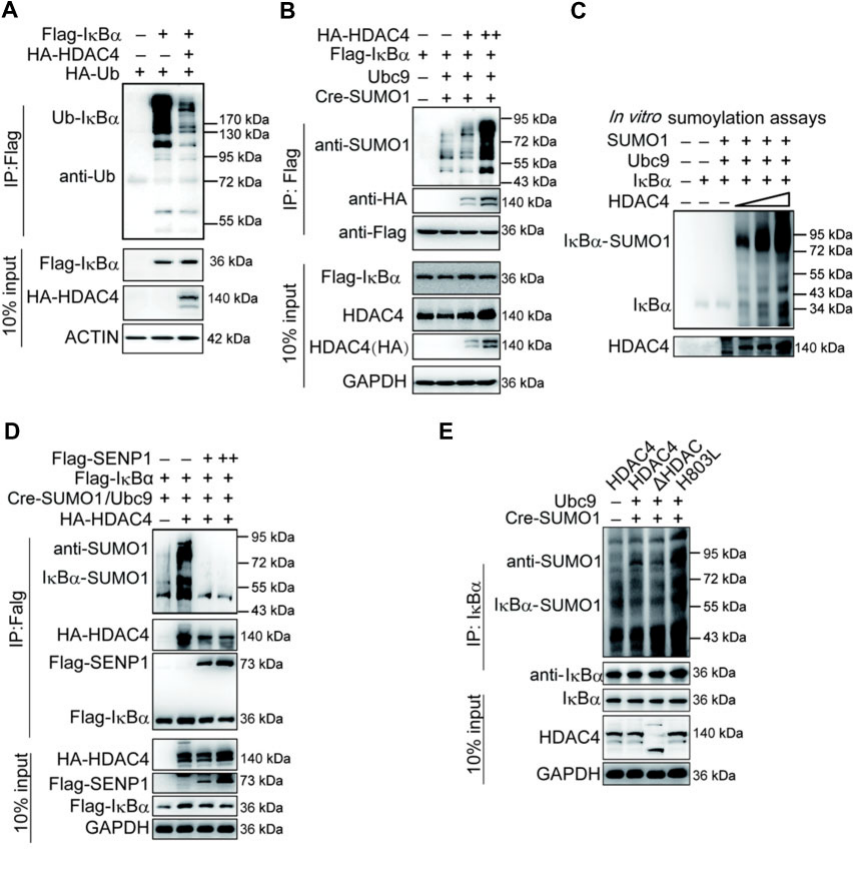
HDAC4 directly modifies the sumoylation of IκBα. (**A**) Overexpression of HDAC4 decreases the ubiquitination of IκBα. HEK293T cells (3 × 10^6^) were transfected with the indicated plasmids. Twenty-four hours after transfection, ubiquitination assays and immunoblotting analysis were performed using the indicated antibodies. (**B**) HDAC4 dose-dependently potentiates the sumoylation of IκBα. HEK293T cells (1 × 10^7^) were cotransfected with Cre-SUMO1, Ubc9, Flag-IκBα, and HDAC4 (1, 5, 10 µg). Sumoylation assays and immunoblotting analysis were performed 36 h after transfection. (**C**) *In vitro* sumoylation of IκBα. The *in vitro* sumoylation assay was performed as described in Materials and methods. The unmodified and SUMO1-modified IκBα proteins are indicated. (**D**) Desumoylation of IκBα by SENP1. The experiments were performed as in **B**, except that the Flag-SENP1 plasmid was used. (**E**) The deacetylase activity site of HDAC4 may not be involved in regulating the sumoylation of IκBα. HDAC4-knockdown HEK293T cells (1 × 10^7^) were transfected with the indicated expression plasmids for WT HDAC4, HDAC4-ΔDAC, or HDAC4-H803L. Sumoylation assays and immunoblotting analysis were performed as in **B**. Data are representative of three independent experiments.

The activation of NF-κB is mediated by signal-induced degradation of IκBα, which allows the active transcription factor to translocate into the nucleus. Sumoylated IκBα was resistant to stimuli-induced proteolysis by ubiquitination (Desterro et al., 1998). We thus determined whether HDAC4 regulated the sumoylation of IκBα and further affected its ubiquitination and degradation. HDAC4 impaired the ubiquitination of IκBα especially the K48-linked ubiquitination of IκBα (Figure 4A; Supplementary Figure S3E). In contrast, knockdown of HDAC4 had opposite effects (Supplementary Figure S3F). Encouragingly, HDAC4 enhanced the SUMO1 modification of IκBα (Figure 4B). To eliminate other confounding factors, we purified IκBα, HDAC4, Ubc9, and SUMO1 using an *in vitro* translation system to perform the *in vitro* sumoylation assays and found that HDAC4 mediated IκBα sumoylation directly (Figure 4C). We also found that SENP1, a desumoylating enzyme, dramatically decreased the sumoylation of IκBα when HDAC4 was overexpressed (Figure 4D). Subsequently, the assay with HDAC4 mutants indicated that loss of deacetylase activity did not affect the sumoylation of IκBα (Figure 4E). Taken together, we concluded that HDAC4 catalyzed the sumoylation of IκBα as a SUMO E3 ligase, which was further supported by the fact that HDAC4 could interact with Ubc9, the SUMO E2 ligase (Supplementary Figure S3G).

**Figure 5:**
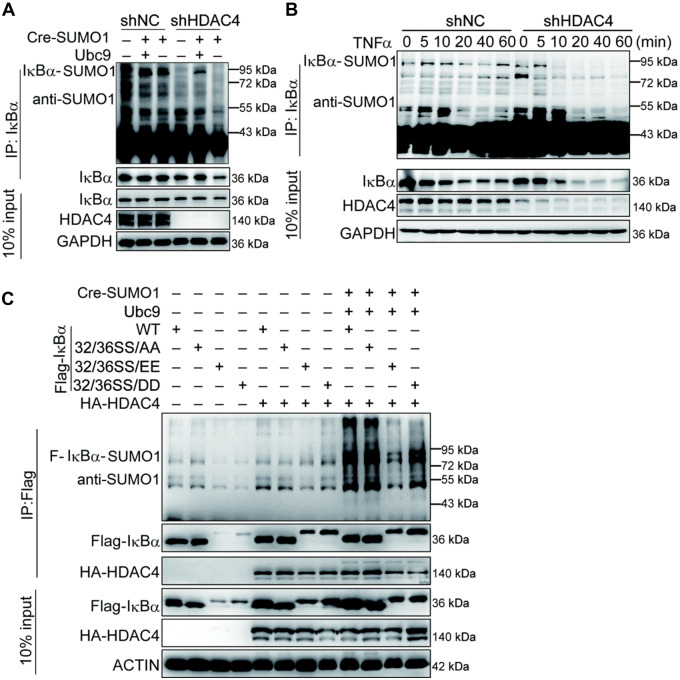
HDAC4 mediates SUMO1 modification of IκBα. (**A**) The knockdown of HDAC4 decreases the sumoylation of IκBα. The control or HDAC4-knockdown HEK293T cells (1 × 10^7^) were transfected with the indicated expression plasmids for 36 h, followed by sumoylation assays and immunoblotting analysis. (**B**) HDAC4 is associated with the endogenous level and SUMO1 modification of IκBα. The WT or HDAC4-stable knockdown HEK293T cells were left untreated or treated with TNFα (20 ng/ml) for the indicated time followed by sumoylation assays and immunoblotting analysis. (**C**) HDAC4 sumoylation of IκBα is slightly impaired by IκBα phosphorylation of residues S32 and S36. The experiments were performed as in **A** except that the indicated plasmids were used. Data are representative of three independent experiments.

The sumoylation of endogenous IκBα was dramatically downregulated in HDAC4-stable knockdown cell lines (Figure 5A). We searched for physiological situations in which the sumoylation of IκBα by HDAC4 occurs in response to TNFα stimulation. Following TNFα stimulation, sumoylated IκBα decreased during the early phase in WT cells and appeared at the late phase (60 min), but the sumoylation of IκBα was significantly decreased and disappeared at the late phase (60 min) in HDAC4-knockdown cells (Figure 5B), which was consistent with the results of Figure 2A and B. It has been reported that ubiquitin modifications of IκBα requires phosphorylation of S32 and S36, but sumoylation is inhibited by phosphorylation (Desterro et al., 1998). Consistently, we validated the results and found that sumoylation and stability of IκBα were inhibited by phosphorylation mimics of IκBα when HDAC4 was present (Figure 5C). These results suggested that the phosphorylation of IκBα at S32/S36 prevented its sumoylation from facilitating degradation.

### HDAC4 C292 is a key site for SUMO E3 ligase activity for catalyzing the sumoylation of IκBα at K21

**Figure 6:**
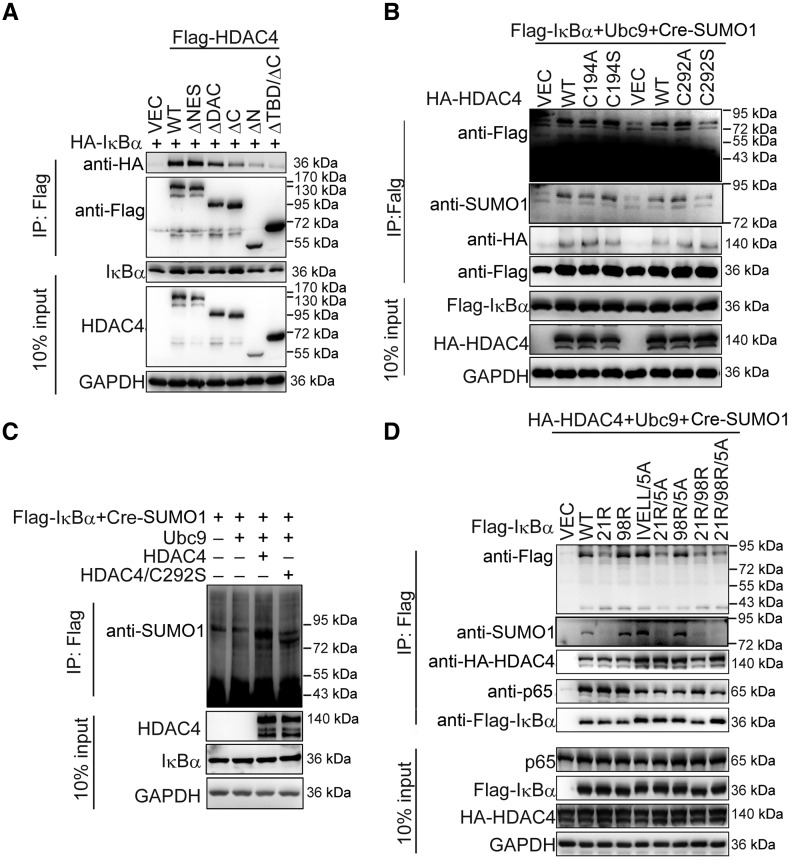
HDAC4 C292 is a key SUMO E3 ligase activity site. (**A**) Domain analysis of HDAC4–IκBα interaction. HEK293T cells (1 × 10^6^) were transiently transfected with the indicated plasmids (3 μg each) for 36 h followed by coIP experiments and immunoblotting analysis with the indicated antibodies. (**B**) HDAC4 C292 as a SUMO E3 ligase activity site. HEK293T cells (1 × 10^7^) were cotransfected with Cre-SUMO1, Ubc9, Flag-IκBα, and the indicated expression plasmids for WT or mutant HDAC4. Sumoylation assays and immunoblotting analysis were performed 36 h after transfection. (**C**) HDAC4 C292S mutant weakens the sumoylation of IκBα in *in vitro* sumoylation assays. The *in vitro* sumoylation assay was performed as described in Materials and methods. (**D**) SUMO1 is mainly conjugated to IκBα K21. The experiments were performed as in **A**. Data are representative of three independent experiments.

To explore which domains of HDAC4 associate with IκBα, we constructed HDAC4 mutants with the deletion of various domains and performed transient transfection and coIP experiments (Supplementary Figure S4A). Results indicated that the transcription binding domain (MEF binding domain, TBD) and C-terminal of HDAC4 were required for the interaction of HDAC4 with IκBα (Figure 6A). Given that the deacetylase capacity of HDAC4 that relies on its C-terminal domain was unessential for the sumoylation of IκBα (Figure 4E), we deduced that the N-terminal TBD of HDAC4 had a potent function for the sumoylation of IκBα.

It has been reported that HDAC4 can sumoylate LXRs or MEF (Zhao et al., 2005; Ghisletti et al., 2007). Bioinformatics analysis found that C194 and C292 reside a motif in the TBD domain that resembles the enzyme activity consensus sites of the classic E3 ligase family (Supplementary Figure S4B). Each of the cysteines was then changed independently to alanine or serine. As shown in Figure 6B, only the C292S mutant could reduce the sumoylation of IκBα by HDAC4. We purified the IκBα, HDAC4, HDAC4/C292S, Ubc9, and SUMO1 using the *in vitro* translation system to perform the *in vitro* sumoylation assays. The results confirmed that C292 was important for the sumoylation of IκBα by HDAC4 (Figure 6C). In summary, these data together suggest that HDAC4 C292 is a key site for SUMO E3 ligase activity and is a potential enzyme activity site for SUMO modification.

We also analyzed the sumoylation sites of IκBα. Besides K21 (Rodriguez et al., 1996), the sumoylation site prediction programs indicated possible sumoylation sites such as K98 and sequence 198–202 (Xue et al., 2006). Mutations harboring K21R resulted in decreased sumoylation of IκBα, suggesting that IκBα was mainly sumoylated at K21 by HDAC4 (Figure 6D).

### Cytoplasmic localization of HDAC4 is essential for the activation of NF-κB signaling

**Figure 7:**
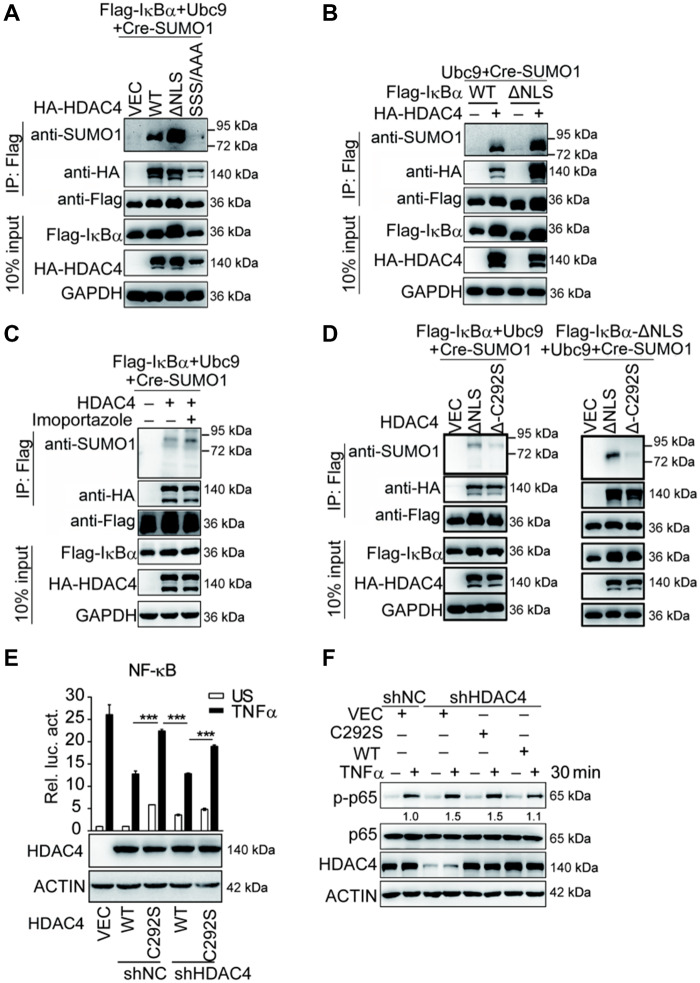
The contribution of HDAC4 C292 to the activation of NF-κB signaling. (**A**) The sumoylation of IκBα requires cytoplasmic localization of HDAC4. HEK293T cells (1 × 10^7^) were cotransfected with Cre-SUMO1, Ubc9, Flag-IκB, and the indicated expression plasmids for WT HDAC4, HDAC4-ΔNLS, or HDAC4-SSS/AAA. Sumoylation assays and immunoblotting analysis were performed 36 h after transfection. (**B**) Sumoylation of IκBα-ΔNLS or IκBα-ΔNES. The experiments were performed as in **A** except that the indicated plasmids were used. (**C**) The inhibition of nuclear localization of HDAC4 enhances the sumoylation of IκBα. The HEK293T cells (1 × 10^7^) were transfected with the indicated expression plasmids for 36 h and then treated with imoportazole (20 nM). The sumoylation assays and immunoblotting analysis were performed as in **A**. (**D**) Effect of HDAC4-ΔNLS-C292S on the sumoylation of IκBα in overexpression sumoylation assays. The experiments were performed as in **A** except that the indicated plasmids were used. (**E**) The enzyme-inactive mutant HDAC4 (C292S) potentiates the promoter activity of NF-κB in HDAC4-stable knockdown HEK293T cells. The control cells (shNC) or HDAC4-stable knockdown HEK293T cells (shHDAC4) reconstituted with WT HDAC4 or HDAC4 C292S mutant were transfected with the NF-κB luciferase reporter plasmid (0.01 µg). Twenty-four hours after transfection, cells were treated with TNFα (20 ng/ml) or left untreated for 10 h before luciferase assays were performed. (**F**) HDAC4 C292S enhances the phosphorylation of NF-κB compared with WT HDAC4 in HDAC4-stable knockdown HEK293T cells. The experiments were performed as in **E**, except that the indicated cells were induced with TNFα (20 ng/ml) for 30 min before the immunoblotting analysis. Data are representative of three independent experiments. Graphs show mean ± SD; *n* = 3. ***P < 0.001.

HDAC4 and IκBα could shuttle between the cytoplasm and nucleus. We constructed a cytoplasmic-localized HDAC4 mutant (HDAC4-ΔNLS) and a nuclear-localized mutant (HDAC4-SSS/AAA) (Supplementary Figure S4C). Sumoylation assay indicated that HDAC4-ΔNLS mutant markedly enhanced the sumoylation of IκBα, whereas HDAC4-SSS/AAA mutant abolished the SUMO modification of IκBα (Figure 7A). Meanwhile, nuclear localization signal (NLS) deletion of IκBα mutant (IκBα-ΔNLS) was located in the cytoplasm (Supplementary Figure S4D). Western blotting analysis demonstrated that IκBα-ΔNLS could be sumoylated by HDAC4 and their interaction was dramatically enhanced compared with the interaction between HDAC4 and WT IκBα protein (Figure 7B). Consistently, importazole, an inhibitor of importin-β transport receptors that make IκBα localize in the cytoplasm (Supplementary Figure S4E), potentiated HDAC4-mediated SUMO1 modification of IκBα (Figure 7C). Furthermore, the double mutant HDAC4-ΔNLS-C292S (Δ-C292S) resulted in a significantly lower SUMO1 modification of IκBα or IκBα-ΔNLS in comparison with the WT HDAC4 (Figure 7D). Taken together, these data suggest that the sumoylation of IκBα occurs in the cytoplasm and highlights that both NLS and C292 are important for the sumoylation of IκBα by HDAC4.

Finally, we investigated the contribution of the enzyme-inactive mutant HDAC4 (C292S) to the transcriptional activity or phosphorylation of NF-κB. We reconstituted WT or C292S mutant into HDAC4-stable knockdown HEK293T cells. The C292S mutant did not inhibit the transcriptional activity and phosphorylation of NF-κB following stimulation with TNFα compared with the WT HDAC4 (Figure 7E and F).

## Discussion

The optimal activation and deactivation of the NF-κB pathway plays crucial roles in controlling several human diseases. Therefore, NF-κB needs to be regulated properly in various manners, including feedback pathways where newly synthesized IκBα serves to shut off the signal (Kracklauer and Schmidt, 2003). In addition, recent studies have revealed that numerous molecules are capable of regulating NF-κB activity negatively (Song et al., 1996; Kovalenko et al., 2003; Sosic et al., 2003; Gao et al., 2004; Liu et al., 2007; Deng et al., 2010; Shembade et al., 2011). In this study, we found that HADC4 also serves as a negative regulator of NF-κB signaling. The ability of HDAC4 to regulate the stability of IκBα is mediated by SUMO E3 ligase activity and the dependence of the modification process on the cytoplasmic localization. Furthermore, the TBD of HDAC4 was required to increase the stability of IκBα, and HDAC4 deficiency caused decreased sumoylation of IκBα compared with WT cells. These results suggest that HDAC4-mediated sumoylation of IκBα is essential for the restriction of the NF-κB signaling pathway.

In our previous work, we showed that HDAC4 could reduce the production of type I IFN in response to various stimuli. HDAC4 can be phosphorylated by TBK1/IKKε directly, which facilitates HDAC4 cytoplasmic localization. Furthermore, IFN-β could stimulate the expression of HDAC4 (Yang et al., 2019). Increasing concentrations of, and cytoplasm localized, HDAC4 could maintain IκBα stability by sumoylation to reduce IFN and inflammatory cytokines and promote the cell to go back to their resting state. Our findings suggest that HDAC4 is a key checkpoint to prevent the overreaction of natural immunity and inflammation.

**Figure 8:**
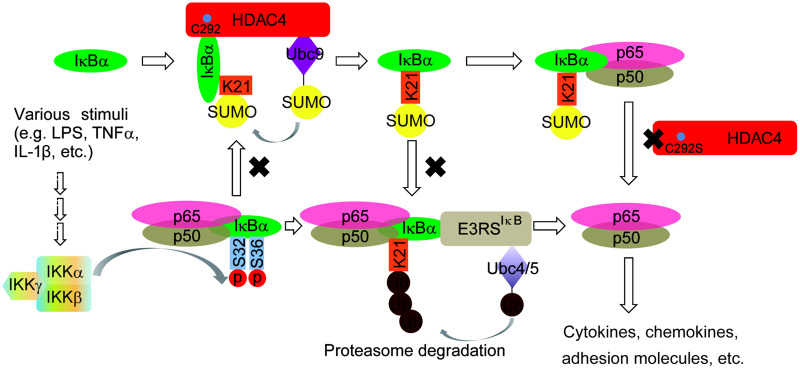
The working model.

IκB is a key regulator as an inhibitor in the NF-κB signaling pathway. NF-κB activation is mediated by the ubiquitylation of phosphorylated IκBα proteins, followed by their proteasomal degradation. Our work and a previous study reported that ubiquitin and SUMO compete for the same target lysine on IκBα, namely, K21, which is also used for ubiquitin conjugation (Desterro et al., 1998). As a result, IκBα modified by SUMO1 cannot be ubiquitinated and is therefore resistant to proteasome-mediated degradation. However, it is unclear if the IκBα modifications cooperate to regulate basal and/or signal-mediated turnover. Several lines of evidence suggest that SUMO modifications achieve this function. First, it has been reported that SUMO1-modified IκBα directly interacts with p50/p65 *in vitro* (Kracklauer and Schmidt, 2003; Lens et al., 2011), which is consistent with our data that endogenous HDAC4 is associated with IκBα, and IκBα is sumoylated and binds to p65 in the resting stage. Then, during the early phase after stimulation, the sumoylation of IκBα was decreased, and IκBα was phosphorylated and followed degradation (Figure 5B). Thus, the desumoylation of IκBα is important for the activation of NF-κB. It will be interesting to identify which desumoylation enzyme(s) is involved in this process. We propose that SENP1 could exercise this function, but further research is needed (Figure 4D). Finally, the interaction between IκBα and NF-κB appeared to be stable in this process (Figure 2). The working model is shown in Figure 8. Our findings suggest that the dynamic sumoylation of IκBα mediated by HDAC4 during stimulation is a cellular moderator to balance the NF-κB activity.

It has been reported that other mechanisms of HDAC4 are at work to regulate immunity and inflammation. Leptin-triggered catecholamine-dependent increases in cAMP signaling resulted in the dephosphorylation of HDAC4, which translocated into the nucleus to impair the acetylation of the histone inactivating the transcription of inflammatory cytokines such as TNFα (Luan et al., 2014). However, only LPS stimulation resulted in HDAC4 localization in the cytoplasm, which is consistent with our results. Thus, under different conditions, HDAC4 employs a different mechanism to regulate the inflammatory pathway negatively. The functional importance of HDAC4 in immunity and inflammation was further supported by our previous data that HDAC4 could reduce the production of type I IFN.

The SUMO E3 ligase activity sites of HDAC4 have not been mapped. We found the TBD and the C-terminal of HDAC4 bind to IκBα. The standard mutation of C292 to serine in TBD significantly reduced the sumoylation of IκBα (Figure 6B), implying that C292 might be an active catalytic site. While not excluding this possibility, there is another enzyme activity site besides C292 for targeting IκBα for sumoylation. In addition, HDAC4 has relatively independent SUMO E3 ligase activity and deacetylase activity. HDAC4 functions as a SUMO E3 ligase in the cytoplasm because a nuclear-localized mutant (HDAC4-SSS/AAA) lost the function to sumoylate IκBα (Figure 7A); HDAC4 in the nucleus functions as deacetylase to diminish histone H4K5 acetylation over TNFα and IL12β promoters (Grozinger and Schreiber, 2000). Thus, both the SUMO E3 ligase and deacetylase activities of HDAC4 play regulatory roles in inflammation in different links.

Due to the functional importance of C292, it is worth considering that the single-nucleotide polymorphisms (SNPs) of HDAC4 C292S (TGT (C)–TCT (S)) are important for inflammation or metabolism; however, there have been no reports of this site. It has been reported that HDAC4 could mediate the sumoylation of MEF2, LXRα, and IκBα, which play key roles in metabolism or inflammation (Gregoire and Yang, 2005; Lee et al., 2009). There was a significant fraction of HDAC4 SNPs associated with body mass index and waist circumference in Caucasians that were also associated with these traits in black and Chinese subjects (Luan et al., 2014). Future efforts should explore the population distribution of this SNP and its association with inflammation or metabolism.

In conclusion, our study provides insights into how HDAC4 acts as a SUMO E3 ligase for IκBα and complex regulatory networks of the NF-κB signaling pathway. In addition, further work will investigate whether deacetylase activity functions and how it might contribute to the regulation of SUMO E3 ligase activity. Given the important roles of class II HDACs in the pathogenesis of cardiac hypertrophy and for the maintenance of insulin sensitivity and energy balance (Zhang et al., 2002; Luan et al., 2014), these enzymes will be a potential target for drug development or disease control in the future.

## Materials and methods

### Reagents and plasmids

Recombinant mouse TNFα, IL-1β, IL-4, M-CSF, and GM-CSF and human TNFα and IFNγ were purchased from R&D Systems. LPS was from Beyotime Biotechnology. Mouse anti-Flag and hyaluronic acid (HA) were from Sigma-Aldrich. Rabbit anti-Flag, HA, HDAC4, HDAC4 phospho-S246, IκBα phospho-S32/36, IKKα/β/γ, mouse anti-IκBα antibody, NF-κB p65 antibody sampler kit, and phospho-IKKα/β (Ser176/180) antibody sampler kit were all purchased from Cell Signaling Technology. Rabbit anti-SUMO1 antibody was kindly provided by Prof. Hanzhong Wang (Wuhan Institute of Virology, Chinese Academy of Sciences). Rabbit anti-IκBα and mouse anti-β-actin antibodies were purchased from Santa Cruz Biotechnology, Inc. Mouse anti-GAPDH antibody was purchased from Proteintech.

HDAC4, IκBα, and p65 were amplified from cDNA of different human or mouse tissues and subsequently cloned into mammalian expression vectors as indicated. The deletion and site-directed mutants of HDAC4 and IκBα, as well as the site-directed mutants of NF-κB, were constructed by a PCR-based approach and then cloned into pXJ40 vectors with Flag- or HA-tag at the N-terminus. All the Ubc9 and Cre-SUMO1 plasmids were kindly provided by Prof. Bing Sun (CAS Center for Excellence in Molecular Cell Science, Chinese Academy of Sciences). The IRF1 promoter luciferase reporter plasmid was provided by Prof. Yanyi Wang. The reporter gene NF-κB was purchased from Clontech. All primers used in this study are described in Supplementary material.

### Cell culture and plasmid transfection

HEK293T and RAW264.7 cells were obtained from American Type Culture Collection and cultured in Dulbecco’s modified Eagle’s medium (Invitrogen) supplemented with 10% fetal bovine serum (FBS) (Invitrogen) at 37°C in a 5% CO_2_ incubator. Cells were stimulated with TNFα (20 ng/ml) or IFNγ (100ng/ml) for the indicated time periods. Plasmids were transfected into cells using Lipofectamine 2000 (Invitrogen) or Lipofectamine 3000 (Invitrogen) following the manufacturer instructions.

### RNA interference experiments

The following siRNAs were targeted for human HDAC4: #1, 5′-CCAAUGUAUUCCAAGCUAA-3′; #2, 5′-GGCGUGGGUUUCAACGUCA-3′. The sequences of siRNAs specific for mouse HDAC4 were: #1, 5′-UCUCUGAUUGAGGCGCAAA-3′; #2, 5′-GGCACAGUUGCAUGAACAU-3′. The siRNAs were transfected using Lipofectamine RNAiMAX reagent (Invitrogen) at a final concentration of 20 nM according to the manufacturer’s instructions. To keep HDAC4 silencing effect for the duration of the test, the cells were split at 24 h posttransfection and transfected with the same siRNA again. Stimulating assay was performed 24 h after the second transfection.

### Luciferase assay

The dual-Luciferase Reporter Assay System (Promega) was used for luciferase assays. Briefly, cells were seeded in 24-well plates (1 × 10^5^ cells per well) and transfected luciferase reporter and pRL-TK plasmids combined with a total of 500 ng of target plasmid or empty control plasmid for 24 h. Subsequently, cells were treated with the indicated reagents or left untreated and luciferase activity was measured as previously described (Chen et al., 2014).

### Lentiviral transduction and establishment of stable cell lines

The lentiviruses system (Bought from addgene, http://www.addgene.org) was used for knockdown and overexpression assays. The lentiviruses were produced as previously described (Guo et al., 2015). In brief, HEK293T cells were cotransfected with the shNC, shHDAC4, or pWPI-HDAC4 plasmid and packaging plasmids psPAX2 and pMD2.G in 4:3:1 ratio. Seventy-two hours after transfection, the supernatant was collected and applied to infect target cells in the presence of polybrene (5 μg/ml). The infected cells were selected by puromycin for 7 days before the conduction of experiments. HEK293T and RAW264.7 cells served for the generation of cell lines with stable knockdown of HDAC4 or stably overexpressing mouse HDAC4 protein, respectively.

### Real-time PCR

Total cellular RNA was isolated with TRIzol (Invitrogen) reagent according to the manufacturer’s protocols. The quantification of specific gene transcripts was analyzed by one-step real-time PCR with the QuantiFast SYBR Green RT-PCR kit (Qiagen). The data were normalized to levels of β-actin mRNA in each individual sample. 2^−ΔΔ^^*Ct*^ method was used to calculate relative expression changes. The primers used are listed as follows: TNFα, sense (GGCGTGGAGCTGAGAGATAAC) and antisense (GGTGTGGGTGAGGAGCACAT); IL-6, sense (TTCTCCACAAGCGCCTTCGGTC) and antisense (TCTGTGTGGGGCGGCTACATCT); IL-8, sense (GAGAGTGATTGAGAGTGGACCAC) and antisense (CACAACCCTCTGCACCCAGTTT); IL-10, sense (GACTTTAAGGGTTACCTGGGTTG) and antisense (TCACATGCGCCTTGATGTCTG); IκBα, sense (CGGGCTGAAGAAGGAGCGGC) and antisense (ACGAGTCCCCGTCCTCGGTG); A20, sense (GCGTTCAGGACACAGACTTG) and antisense (GCAAAGCCCCGTTTCAACAA); GAPDH, sense (GACAAGCTTCCCGTTCTCAG) and antisense (GAGTCAACGGATTTGGTCGT); mTNFα, sense (GACGTGGAACTGGCAGAAGAG) and antisense (TTGGTGGTTTGTGAGTGTGAG); mIL-6, sense (TAGTCCTTCCTACCCCAATTTCC) and antisense (TTGGTCCTTAGCCACTCCTTC); mIL-10, sense (GGACAACATACTGCTAACCG) and antisense (TTCATGGCCTTGTAGACACC); mIκBα, sense (TGAAGGACGAGGAGTACGAGC) and antisense (TGCAGGAACGAGTCTCCGT); mA20, sense (ACCATGCACCGATACACGC) and antisense (AGCCACGAGCTTCCTGACT); mβ-actin, sense (AGTGTGACGTTGACATCCGT) and antisense (GCAGCTCAGTAACAGTCCGC).

### Preparation of bone marrow-derived macrophages and BMDCs

Bone marrow cells were isolated from the femurs and tibiae of mice. For the preparation of bone marrow-derived macrophages (BMDMs), bone marrow cells were cultured in RPMI-1640 containing 10% FBS, 10 mM HEPES (pH 7.4), and 10 ng/ml M-CSF. Twenty-four hours later, nonadherent cells were transferred to a new flask and cultured for another 4 days. For the preparation of BMDCs, bone marrow cells were cultured for 9 days in RPMI-1640 containing 10% FBS, 10 mM HEPES (pH 7.4), 10 ng/ml GM-CSF, and 5 ng/ml IL-4. Mature BMDMs and BMDCs were harvested by collagenase (Roche) digestion and cultured on 6-well or 12-well plates for experiments.

### Detection of cytokine production

The concentration of TNFα and IL-6 in culture supernatants was measured with a mouse TNFα ELISA kit or a mouse IL-6 ELISA kit (R&D Systems). See the instructions for details of the experiment.

### Western blotting and coIP

Whole-cell lysates for both western blotting and IP were prepared 48 h post-transient transfection in an IP lysis buffer containing 50 mM Tris, pH 7.5, 1 mM EGTA, 1 mM EDTA, 1% Triton X-100, 150 mM NaCl, 100 µM phenylmethylsulfonyl fluoride, and Complete™ protease inhibitors (Roche Applied Science) for 30 min in 4°C. Cell lysates were centrifuged at 14000× *g* for 10 min at 4°C and quantified using the Bradford method. For western blotting, the supernatants were recovered and boiled in loading buffer. For IP, the supernatants were collected and mixed with Protein G-agarose (Millipore) and various antibodies for 4 h at 4°C. Protein G agarose-bound immune complexes were then eluted and subjected to western blotting analysis. The proteins were visualized using suitable horseradish peroxidase-conjugated secondary antibodies (Jackson Immuno Research) and SuperSignal-Femto chemiluminescent substrate (Pierce).

### Immunofluorescent confocal microscopy

HEK293T cells were plated on glass coverslips and transfected with the indicated plasmids by Lipofectamine 2000 (Invitrogen). At 24 h after transfection, the cells were treated with the indicated reagents or left untreated for the indicated time points followed by fixation, permeabilization, and blockade. Next, cells were incubated with the appropriate primary antibodies for 1 h and Alexa Fluor 561/488-conjugated secondary antibodies (Invitrogen) were added for 1 h. Then the cells were washed three times with PBS and stained with DAPI (Invitrogen). The cells were observed with a Perkin–Elmer UltraView Vox confocal microscope under a 60× oil objective.

### In vitro sumoylation assays

The tested proteins HDAC4 or HDAC4/C292S, Ubc9, SUMO1, and IκBα in Figures 4C and 6C were *in vitro* translated with a TNT Quick-coupled Transcription/Translation Systems kit (Promega) following instructions of the manufacturer. SAE1/SAE2 (100 ng), Ubc9 (50 ng), SUMO1 (1 μg), IκBα (1 μg), and HDAC4 (1–4 μg) were added to the buffer (50 mM Tris–HCl, pH 7.5, 2.5 mM Mg^2+^, 2.5 mM DTT, and 2 mM ATP). The mix was fractionated by SDS–PAGE and analyzed by immunoblotting with anti-Flag antibody.

### Statistics

The results are quantifications from multiple independent experiments and are described in each corresponding figure legend. Coprecipitation efficiency was measured with ImageJ program. GraphPad Prism 5 software was used for all statistical analyses. Quantitative data displayed as histograms are presented as mean ± SD (represented as error bars). Data were analyzed using a Student’s unpaired *t*-test or multiple *t*-test. *P*-values were calculated, and statistical significance was reported with asterisk (*P < 0.05, **P < 0.01, ***P < 0.001).

## Supplementary material

Supplementary material is available at *Journal of Molecular Cell Biology* online.

## Supplementary Material

mjaa043_Supplementary_Data

## References

[mjaa043-B1] Aung H.T. , Schroder K., Himes S.R., et al (2006). LPS regulates proinflammatory gene expression in macrophages by altering histone deacetylase expression. FASEB J. 20, 1315–1327. 16816106 10.1096/fj.05-5360com

[mjaa043-B2] Baeuerle P.A. , Baichwal V.R. (1997). NF-κB as a frequent target for immunosuppressive and anti-inflammatory molecules. Adv. Immunol. 65, 111–137. 9238509

[mjaa043-B3] Chen H. , Pei R., Zhu W., et al (2014). An alternative splicing isoform of MITA antagonizes MITA-mediated induction of type I IFNs. J. Immunol. 192, 1162–1170. 24391220 10.4049/jimmunol.1300798

[mjaa043-B4] Chen L.F. , Mu Y., Greene W.C. (2002). Acetylation of RelA at discrete sites regulates distinct nuclear functions of NF-κB. EMBO J. 21, 6539–6548. 12456660 10.1093/emboj/cdf660 PMC136963

[mjaa043-B5] Choi S.J. , Lee H.C., Kim J.H., et al (2016). HDAC6 regulates cellular viral RNA sensing by deacetylation of RIG-I. EMBO J. 35, 429–442. 26746851 10.15252/embj.201592586 PMC4755110

[mjaa043-B6] de Zoeten E.F. , Wang L., Sai H., et al (2010). Inhibition of HDAC9 increases T regulatory cell function and prevents colitis in mice. Gastroenterology 138, 583–594. 19879272 10.1053/j.gastro.2009.10.037 PMC3369426

[mjaa043-B7] Deng N. , Ye Y., Wang W., et al (2010). Dishevelled interacts with p65 and acts as a repressor of NF-κB-mediated transcription. Cell Res. 20, 1117–1127. 20628365 10.1038/cr.2010.108

[mjaa043-B8] Desterro J.M. , Rodriguez M.S., Hay R.T. (1998). SUMO-1 modification of IκBα inhibits NF-κB activation. Mol. Cell 2, 233–239. 9734360 10.1016/s1097-2765(00)80133-1

[mjaa043-B9] Foster S.L. , Hargreaves D.C., Medzhitov R. (2007). Gene-specific control of inflammation by TLR-induced chromatin modifications. Nature 447, 972–978. 17538624 10.1038/nature05836

[mjaa043-B10] Gao H. , Sun Y., Wu Y., et al (2004). Identification of β-arrestin2 as a G protein-coupled receptor-stimulated regulator of NF-κB pathways. Mol. Cell 14, 303–317. 15125834 10.1016/s1097-2765(04)00216-3

[mjaa043-B11] Ghisletti S. , Huang W., Ogawa S., et al (2007). Parallel SUMOylation-dependent pathways mediate gene- and signal-specific transrepression by LXRs and PPARγ. Mol. Cell 25, 57–70. 17218271 10.1016/j.molcel.2006.11.022 PMC1850387

[mjaa043-B12] Gregoire S. , Yang X.J. (2005). Association with class IIa histone deacetylases upregulates the sumoylation of MEF2 transcription factors. Mol. Cell. Biol. 25, 2273–2287. 15743823 10.1128/MCB.25.6.2273-2287.2005 PMC1061617

[mjaa043-B13] Grivennikov S.I. , Greten F.R., Karin M. (2010). Immunity, inflammation, and cancer. Cell 140, 883–899. 20303878 10.1016/j.cell.2010.01.025 PMC2866629

[mjaa043-B14] Grozinger C.M. , Schreiber S.L. (2000). Regulation of histone deacetylase 4 and 5 and transcriptional activity by 14-3-3-dependent cellular localization. Proc. Natl Acad. Sci. USA 97, 7835–7840. 10869435 10.1073/pnas.140199597 PMC16631

[mjaa043-B15] Guo M. , Pei R.J., Yang Q., et al (2015). Phosphatidylserine-specific phospholipase A1 involved in hepatitis C virus assembly through NS2 complex formation. J. Virol. 89, 2367–2377. 25505071 10.1128/JVI.02982-14 PMC4338883

[mjaa043-B16] Harhaj E.W. , Dixit V.M. (2011). Deubiquitinases in the regulation of NF-κB signaling. Cell Res. 21, 22–39. 21119682 10.1038/cr.2010.166 PMC3075605

[mjaa043-B17] Karin M. , Ben-Neriah Y. (2000). Phosphorylation meets ubiquitination: the control of NF-κB activity. Annu. Rev. Immunol. 18, 621–663. 10837071 10.1146/annurev.immunol.18.1.621

[mjaa043-B18] Kovalenko A. , Chable-Bessia C., Cantarella G., et al (2003). The tumour suppressor CYLD negatively regulates NF-κB signalling by deubiquitination. Nature 424, 801–805. 12917691 10.1038/nature01802

[mjaa043-B19] Kracklauer M.P. , Schmidt C. (2003). At the crossroads of SUMO and NF-κB. Mol. Cancer 2, 39. 14613580 10.1186/1476-4598-2-39 PMC280695

[mjaa043-B20] Lee J.H. , Park S.M., Kim O.S., et al (2009). Differential SUMOylation of LXRα and LXRβ mediates transrepression of STAT1 inflammatory signaling in IFN-γ-stimulated brain astrocytes. Mol. Cell 35, 806–817. 19782030 10.1016/j.molcel.2009.07.021

[mjaa043-B21] Lens Z. , Dewitte F., Van Lint C., et al (2011). Purification of SUMO-1 modified IκBα and complex formation with NF-κB. Protein Expr. Purif. 80, 211–216. 21708266 10.1016/j.pep.2011.06.009

[mjaa043-B22] Li X. , Zhang Q., Ding Y., et al (2016). Methyltransferase Dnmt3a upregulates HDAC9 to deacetylate the kinase TBK1 for activation of antiviral innate immunity. Nat. Immunol. 17, 806–815. 27240213 10.1038/ni.3464

[mjaa043-B23] Liu B. , Yang Y., Chernishof V., et al (2007). Proinflammatory stimuli induce IKKα-mediated phosphorylation of PIAS1 to restrict inflammation and immunity. Cell 129, 903–914. 17540171 10.1016/j.cell.2007.03.056

[mjaa043-B24] Luan B. , Goodarzi M.O., Phillips N.G., et al (2014). Leptin-mediated increases in catecholamine signaling reduce adipose tissue inflammation via activation of macrophage HDAC4. Cell Metab. 19, 1058–1065. 24768298 10.1016/j.cmet.2014.03.024 PMC4207085

[mjaa043-B25] McKinsey T.A. , Zhang C.L., Lu J., et al (2000). Signal-dependent nuclear export of a histone deacetylase regulates muscle differentiation. Nature 408, 106–111. 11081517 10.1038/35040593 PMC4459600

[mjaa043-B26] Meng J. , Liu X., Zhang P., et al (2016). Rb selectively inhibits innate IFN-β production by enhancing deacetylation of IFN-β promoter through HDAC1 and HDAC8. J. Autoimmun. 73, 42–53. 27267461 10.1016/j.jaut.2016.05.012

[mjaa043-B27] Miska E.A. , Karlsson C., Langley E., et al (1999). HDAC4 deacetylase associates with and represses the MEF2 transcription factor. EMBO J. 18, 5099–5107. 10487761 10.1093/emboj/18.18.5099 PMC1171580

[mjaa043-B28] Navarro M.N. , Goebel J., Feijoo-Carnero C., et al (2011). Phosphoproteomic analysis reveals an intrinsic pathway for the regulation of histone deacetylase 7 that controls the function of cytotoxic T lymphocytes. Nat. Immunol. 12, 352–361. 21399638 10.1038/ni.2008 PMC3110993

[mjaa043-B29] Oeckinghaus A. , Hayden M.S., Ghosh S. (2011). Crosstalk in NF-κB signaling pathways. Nat. Immunol. 12, 695–708. 21772278 10.1038/ni.2065

[mjaa043-B30] Rodriguez M.S. , Wright J., Thompson J., et al (1996). Identification of lysine residues required for signal-induced ubiquitination and degradation of IκB-α in vivo. Oncogene 12, 2425–2435. 8649784

[mjaa043-B31] Shembade N. , Pujari R., Harhaj N.S., et al (2011). The kinase IKKα inhibits activation of the transcription factor NF-κB by phosphorylating the regulatory molecule TAX1BP1. Nat. Immunol. 12, 834–843. 21765415 10.1038/ni.2066 PMC3205447

[mjaa043-B32] Song H.Y. , Rothe M., Goeddel D.V. (1996). The tumor necrosis factor-inducible zinc finger protein A20 interacts with TRAF1/TRAF2 and inhibits NF-κB activation. Proc. Natl Acad. Sci. USA 93, 6721–6725. 8692885 10.1073/pnas.93.13.6721 PMC39093

[mjaa043-B33] Sosic D. , Richardson J.A., Yu K., et al (2003). Twist regulates cytokine gene expression through a negative feedback loop that represses NF-κB activity. Cell 112, 169–180. 12553906 10.1016/s0092-8674(03)00002-3

[mjaa043-B34] Sun S.C. (2008). Deubiquitylation and regulation of the immune response. Nat. Rev. Immunol. 8, 501–511. 18535581 10.1038/nri2337 PMC5763493

[mjaa043-B35] Sun S.C. , Ganchi P.A., Ballard D.W., et al (1993). NF-κB controls expression of inhibitor IκBα: evidence for an inducible autoregulatory pathway. Science 259, 1912–1915. 8096091 10.1126/science.8096091

[mjaa043-B36] Traenckner E.B. , Pahl H.L., Henkel T., et al (1995). Phosphorylation of human IκB-α on serines 32 and 36 controls IκB-α proteolysis and NF-κB activation in response to diverse stimuli. EMBO J. 14, 2876–2883. 7796813 10.1002/j.1460-2075.1995.tb07287.x PMC398406

[mjaa043-B37] Vallabhapurapu S. , Karin M. (2009). Regulation and function of NF-κB transcription factors in the immune system. Annu. Rev. Immunol. 27, 693–733. 19302050 10.1146/annurev.immunol.021908.132641

[mjaa043-B38] Xue Y. , Zhou F., Fu C., et al (2006). SUMOsp: a web server for sumoylation site prediction. Nucleic Acids Res. 34, W254–W257. 16845005 10.1093/nar/gkl207 PMC1538802

[mjaa043-B39] Yamaguchi T. , Cubizolles F., Zhang Y., et al (2010). Histone deacetylases 1 and 2 act in concert to promote the G1-to-S progression. Genes Dev. 24, 455–469. 20194438 10.1101/gad.552310 PMC2827841

[mjaa043-B40] Yang Q. , Tang J., Pei R., et al (2019). Host HDAC4 regulates the antiviral response by inhibiting the phosphorylation of IRF3. J. Mol. Cell Biol. 11, 158–169. 29800227 10.1093/jmcb/mjy035 PMC6734143

[mjaa043-B41] Zhang C.L. , McKinsey T.A., Chang S., et al (2002). Class II histone deacetylases act as signal-responsive repressors of cardiac hypertrophy. Cell 110, 479–488. 12202037 10.1016/s0092-8674(02)00861-9 PMC4459650

[mjaa043-B42] Zhao X. , Sternsdorf T., Bolger T.A., et al (2005). Regulation of MEF2 by histone deacetylase 4- and SIRT1 deacetylase-mediated lysine modifications. Mol. Cell. Biol. 25, 8456–8464. 16166628 10.1128/MCB.25.19.8456-8464.2005 PMC1265742

