# Supplementary material for: Histone deacetylase 4 inhibits NF-κB activation by facilitating IκBα sumoylation
Source: J Mol Cell Biol. 2021 Feb 4;12(12):933–45. doi: 10.1093/jmcb/mjaa043 (PMC7948076; doi:10.1093/jmcb/mjaa043)
Supplement: mjaa043_Supplementary_Data [file mjaa043_supplementary_data.pdf]

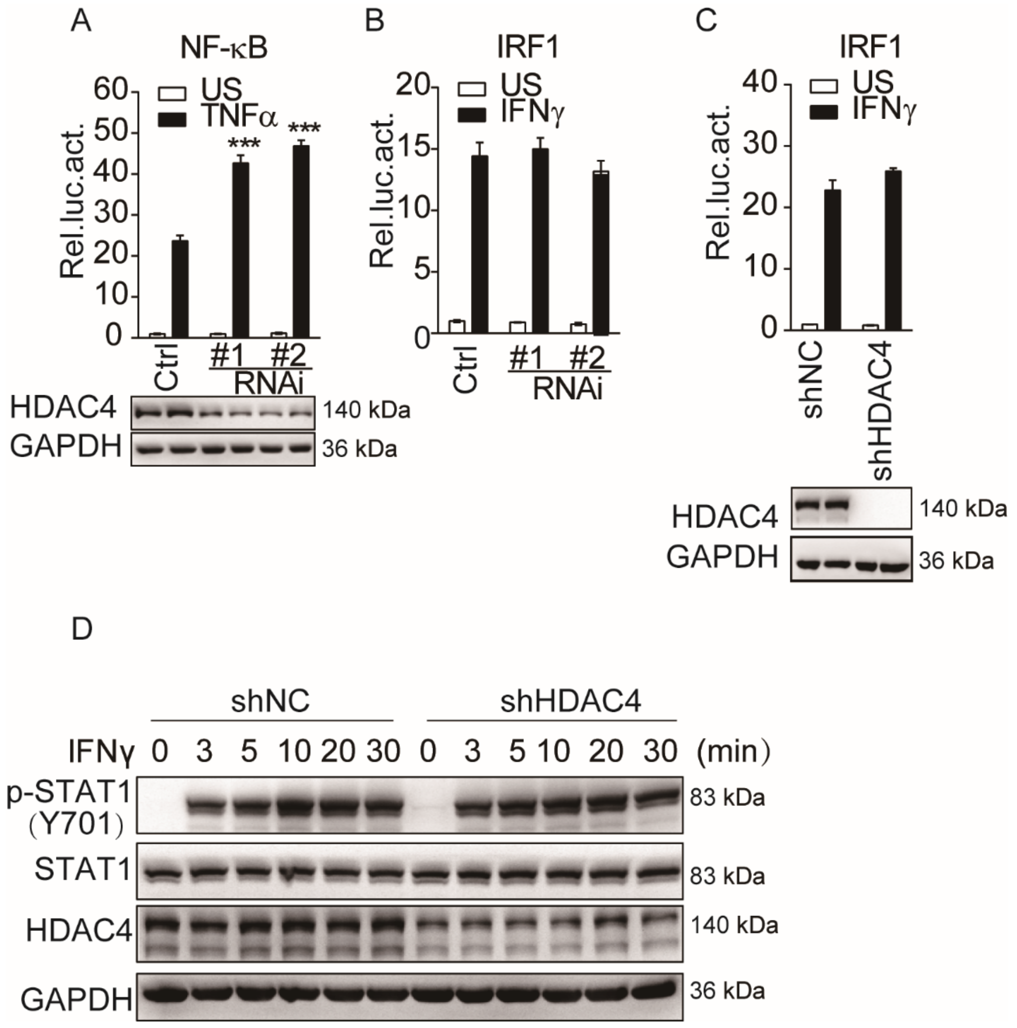

Supplementary Figure 1. Effect of HDAC4 knockdown on IFNγ-induced activation of the IRF1 promoter. (A) Effect of HDAC4-RNAi on TNFα-triggered NF-κB or activation in HEK293T cells. Cells (1×10^5) were transfected with different siRNAs as indicated and then transfected with the NF-κB reporter plasmid (0.01 μg). Twenty-four hours after transfection, cells were treated with TNFα (20 ng/mL) or left untreated for 10 h before luciferase assays were performed. **(B)** Effect of HDAC4 knockdown via siRNA or **(C)** the stable knockdown cell-lines on IFNγ-induced activation of the IRF1 promoter. The reporter assays were performed as in A except that the IRF1 promoter reporter plasmid (0.1 μg) was used and the transfected cells were treated with IFNγ (100 ng/mL). **(D)** Effects of HDAC4 knockdown on IFNγ-triggered activation of STAT1. The indicated cells (5×10^5) were left untreated or treated with IFNγ (100 ng/mL) for the indicated times. Immunoblot analysis was performed with the indicated antibodies.

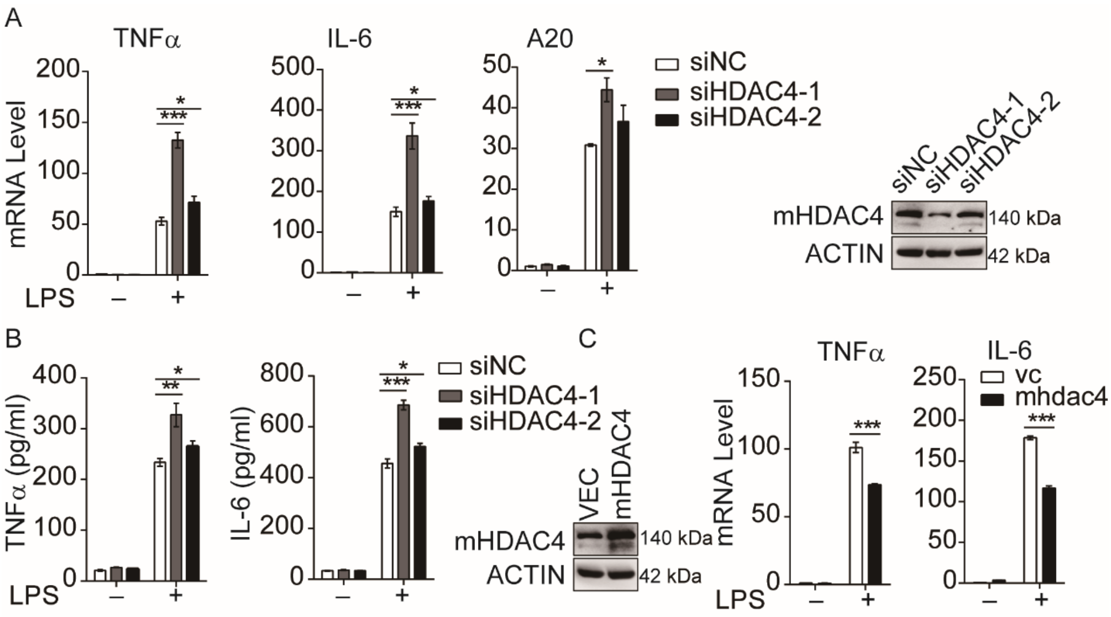

Supplementary Figure 2. HDAC4 potentiates LPS-triggered NF-κB signaling in mouse macrophage cells. (A) Efficiencies of mHDAC4-RNAi siRNAs for HDAC4 levels and effect of HDAC4-RNAi on LPS-triggered transcription of *TNFα*, *IL-6* and *A20* genes in mouse RAW264.7 cell line. RAW264.7 cells (8×10^5) were transfected with control or the indicated siRNAs (20 μM each) for 48 h. Cell lysates were analyzed by immunoblot with anti-HDAC4 or anti–β-actin. The control or HDAC4 knockdown cells (2×10^5) were treated with LPS (100 ng/mL) for the indicated times or left untreated, and then total RNA was prepared for quantitative real-time PCR. The results were normalized to *ACTIN* transcription. **(B)** Effects of HDAC4-RNAi on LPS-induced cytokines of TNFα and IL-6 in mouse RAW264.7 cell line. Cells (4×10^5) were treated with LPS for 6 h, and then the medium was collected for ELISA. **(C)** RAW264.7 cells served for the generation of cell lines stably overexpressing mouse HDAC4 protein. Effect of overexpression of HDAC4 on LPS-triggered transcription of *TNFα* and *IL-6* genes in mouse RAW264.7 cell line. The experiments were performed as in C except that the cell lines stably overexpressing mouse HDAC4 proteins was used and treated with LPS (100 ng/mL). Data are representative of three independent experiments. Graphs show mean ± SD; $n = 3$. *$*P < 0.05$; $**P < 0.01$; $***P < 0.001$.*

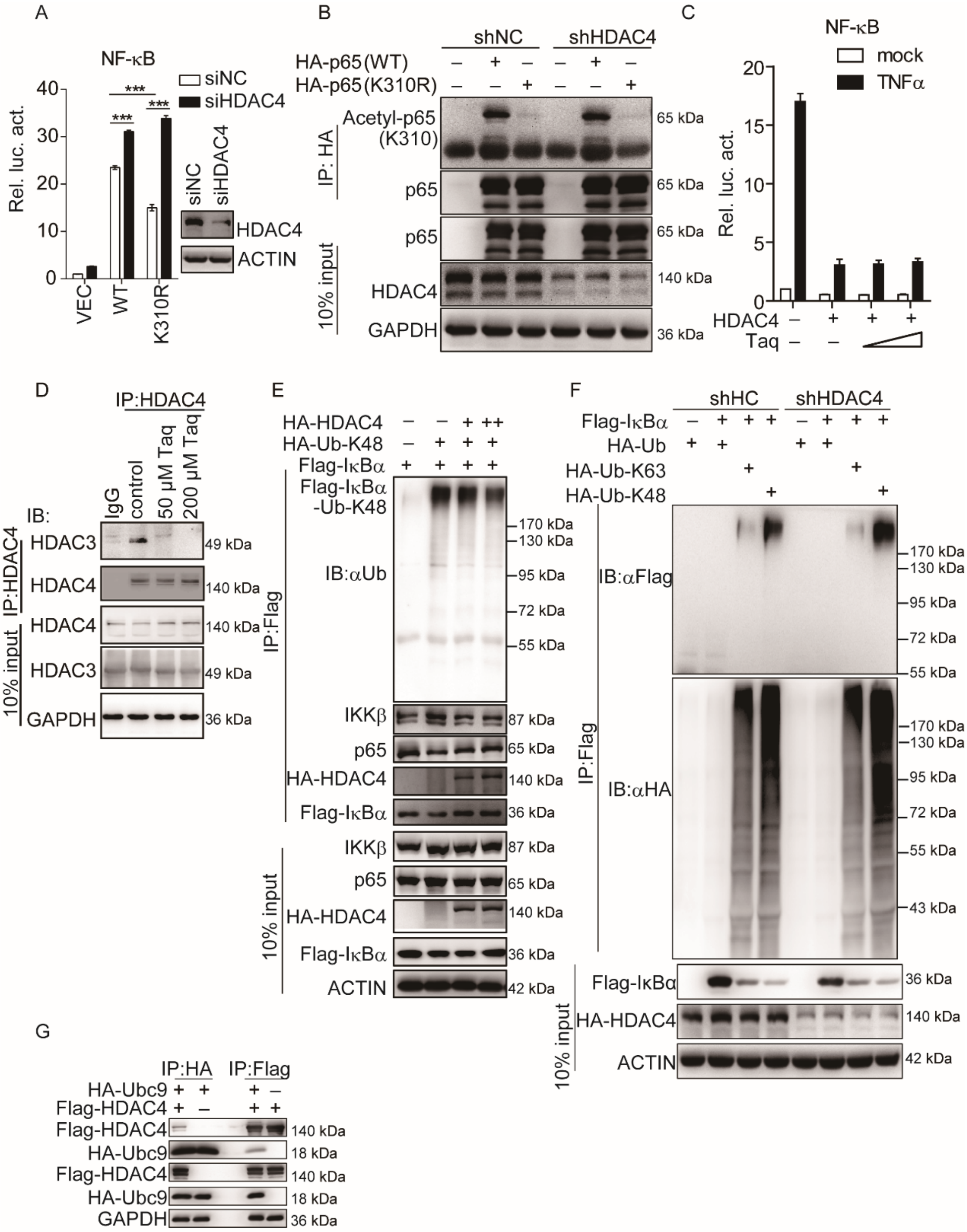

Supplementary Figure 3. **(A)** Effect of HDAC4 on p65- or p65 K310R-triggered NF-κB activation in HDAC4 stable knockdown HEK293T cells. The cells (1×10^5) from stable cell lines were transfected with the NF-κB reporter plasmid (0.01 μg) together with the wild-type p65 or p65 K310R mutant (0.3 μg). Twenty-four hours after transfection, luciferase activity was measured as described previously. **(B)** Analyses of acetylation status of wild-type p65 and the K310R mutant of p65. Whole-cell extracts from HDAC4 stable knockdown HEK293T cells transfected for 24 h with expression vectors encoding wild-type p65 or the K310R mutant of p65 were prepared. The immunoprecipitated proteins were analyzed

by immunoblot with the indicated antibodies. **(C)** Luciferase assay analysis of NF-κB promoter activity in HEK293T cells transfected for 24 h with a luciferase reporter plasmid, plus the HDAC4-specific inhibitor Tasquinimod (wedge:50 and 200 μM), and then left uninfected or infected for 8 h with TNFα (20 ng/mL). **(D)** Tasquinimod inhibits HDAC4 binding to HDAC3 in HEK-293 cells. **(E)** HDAC4 weakens the K48-linked polyubiquitination of IκBα in a dose-dependent manner. HEK293T cells (3×10^6) were transfected with the indicated plasmids. Twenty-four hours after transfection, ubiquitination assays and immunoblotting analysis were performed with the indicated antibodies. **(F)** Knockdown of HDAC4 enhances the K48-linked polyubiquitination of IκBα. The control or HDAC4 knockdown cells (3×10^6) were transfected with the indicated plasmids. Twenty-four hours after transfection, cell lysates were immunoprecipitated with anti-Flag antibody and the immunoprecipitates were analyzed by immunoblots with anti- IκBα. Data are representative of three independent experiments. (**G**) HDAC4 interacts with Ubc9 in overexpression system. HEK293T cells (1×10^6) were transfected with the indicated plasmids (3 μg each) for 36 h followed by co-immunoprecipitation experiments and immunoblotting analysis with the indicated antibodies. Data are representative of three independent experiments. Graphs show mean ± SD; n = 3. ***P < 0.001.

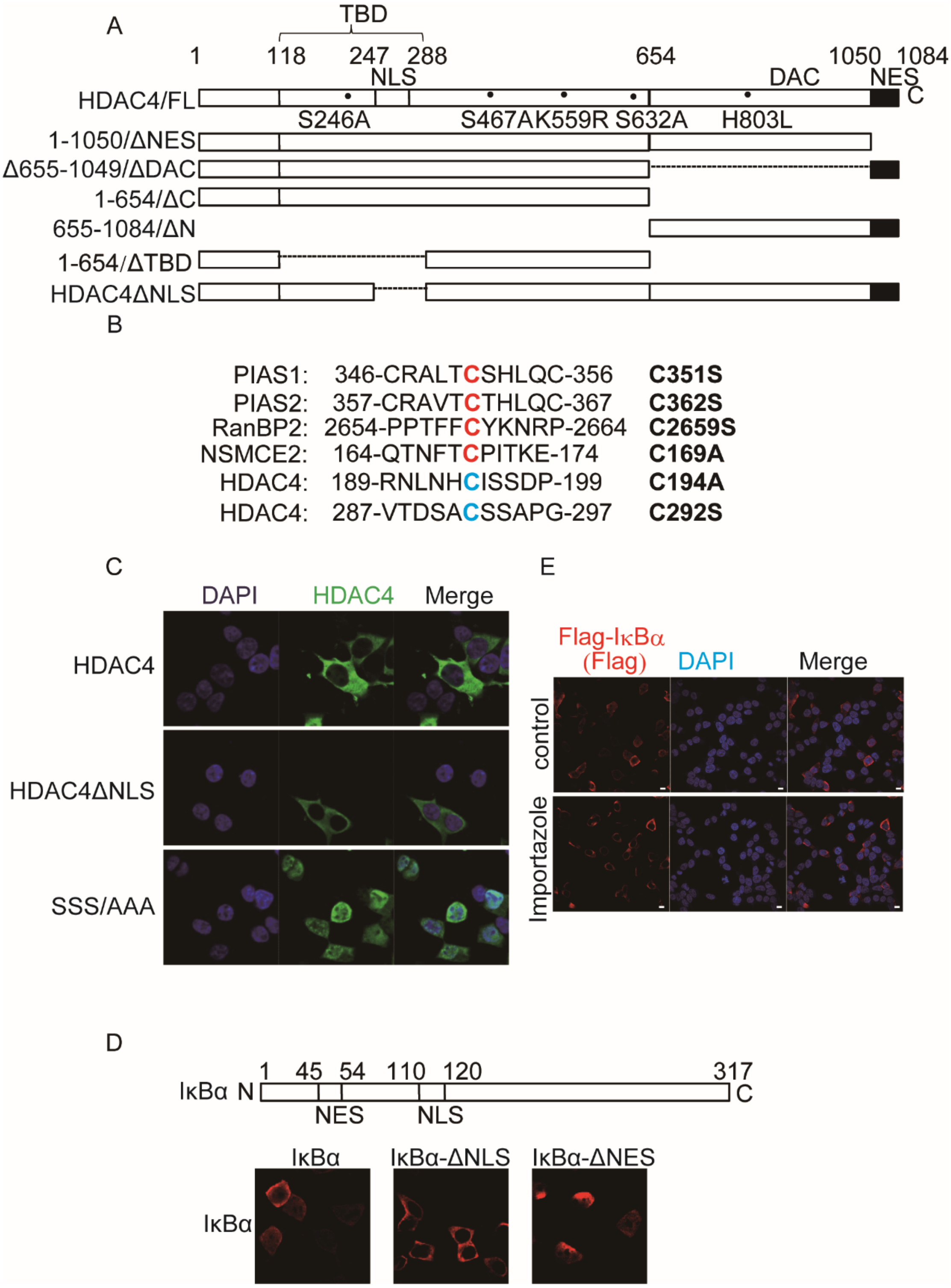

Supplementary Figure 4. (A) Strategy for the generation of HDAC4 and its mutants. Structure of wild-type HDAC4 (top), showing the transcription binding domain (TBD), nuclear localisation signal (NLS), deacetylation domain (DAC), and nuclear export signal (18) and site-specific mutants incuding mutations of phosphorylation sites (S246A, S467A and S632A, named SSS/AAA) and mutation of deacetylase activity site (H803L), and truncation and deletion mutants of HDAC4 various domains (below). **(B)** Sequencing alignment of classic SUMO E3 ligase (PIAS1, PIAS2, RanBP2 and NSMCE2) with HDAC4. The red "C" means the enzyme center of the ligase. The blue "C" means predicted enzyme

activity centers. **(C)** The localization of HDAC4-△NLS and HDAC4-SSS/AAA. HEK293T cells (5×10^4) were transfected with the indicated plasmids (0.5 μg) for 24 h. Cells were fixed with 4% (wt/vol) paraformaldehyde and subjected for immunofluorescence experiments and confocal microscopy analysis. **(D)** The localization of IκBα was analyzed by confocal with Importazole (20nmol/L) treatment or not. (**E**) The localization of IκBα-△NLS and IκBα-△NES was analyzed by confocal. The experiments were performed as in B.
